# (Ferrocene­carboxyl­ato-κ*O*)triphenyl­tin(IV)

**DOI:** 10.1107/S1600536810022488

**Published:** 2010-06-18

**Authors:** Youzhu Yu, Chengchen Zhu, Jianping Huang, Qingchao Jia, Nan Zhang

**Affiliations:** a Department of Chemistry and Environmental Engineering, Anyang Institute of Technology, Henan 455000, People’s Republic of China; b College of Chemistry and Chemical Engineering, Liaocheng University, Shandong 252059, People’s Republic of China

## Abstract

In the title compound, [FeSn(C_5_H_5_)(C_6_H_5_)_3_(C_6_H_4_O_2_)], the Sn^IV^ atom displays a distorted tetra­hedral coordination geometry, provided by one O atom of the monodentate ferrocene­carboxyl­ate ligand [Sn—O = 2.079 (2) Å] and by three C atoms of the three phenyl groups [average Sn—C = 2.130 (4) Å]. No classic hydrogen bonds or inter­molecular inter­actions are observed in the crystal.

## Related literature

For related structures, see: Kim *et al.* (2007 ▶); Tao *et al.* (1997 ▶); Wang *et al.* (2007 ▶); Zhang *et al.* (2002 ▶); Zheng, Ma, Su *et al.* (2004 ▶); Zheng, Ma, Yang *et al.* (2004 ▶); Yu *et al.* (2010 ▶).
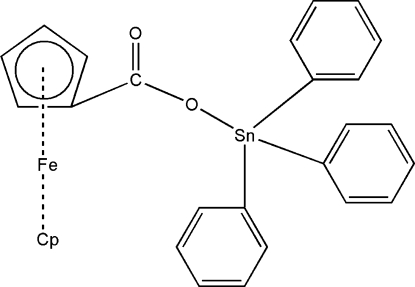


## Experimental

### 

#### Crystal data

[FeSn(C_5_H_5_)(C_6_H_5_)_3_(C_6_H_4_O_2_)]
*M* *_r_* = 579.02
Triclinic, 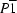

*a* = 10.1012 (15) Å
*b* = 11.4066 (18) Å
*c* = 11.6741 (19) Å
α = 98.034 (1)°
β = 106.107 (2)°
γ = 106.082 (2)°
*V* = 1207.1 (3) Å^3^
*Z* = 2
Mo *K*α radiation
μ = 1.66 mm^−1^
*T* = 298 K
0.50 × 0.40 × 0.38 mm

#### Data collection

Bruker SMART 1000 CCD diffractometer
Absorption correction: multi-scan (*SADABS*; Sheldrick, 1996 ▶) *T*_min_ = 0.491, *T*_max_ = 0.571
6299 measured reflections
4182 independent reflections
3539 reflections with *I* > 2σ(*I*)
*R*_int_ = 0.021

#### Refinement

*R*[*F* ^2^ > 2σ(*F* ^2^)] = 0.030
*wR*(*F* ^2^) = 0.089
*S* = 1.01
4182 reflections
298 parameters
H-atom parameters constrained
Δρ_max_ = 0.46 e Å^−3^
Δρ_min_ = −0.70 e Å^−3^

### 

Data collection: *SMART* (Siemens, 1996 ▶); cell refinement: *SAINT* (Siemens, 1996 ▶); data reduction: *SAINT*; program(s) used to solve structure: *SHELXS97* (Sheldrick, 2008 ▶); program(s) used to refine structure: *SHELXL97* (Sheldrick, 2008 ▶); molecular graphics: *SHELXTL* (Sheldrick, 2008 ▶); software used to prepare material for publication: *SHELXTL*.

## Supplementary Material

Crystal structure: contains datablocks I, global. DOI: 10.1107/S1600536810022488/zq2044sup1.cif

Structure factors: contains datablocks I. DOI: 10.1107/S1600536810022488/zq2044Isup2.hkl

Additional supplementary materials:  crystallographic information; 3D view; checkCIF report

## Figures and Tables

**Fig. 1.**
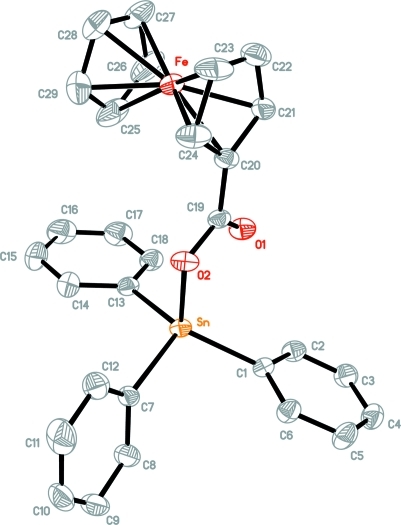
The molecular structure of the title compound, with atom labels and 30% probability displacement ellipsoids.

## supplementary crystallographic information

### Comment

In continuation of structural and biological activity studies of organotin compounds (Zheng, Ma, Su *et al.*, 2004; Zheng, Ma, Yang *et al.*, 2004), we report here the synthesis and the crystal structure of the title compound.

In the crystal structure of the title compound, [Sn(C_11_H_9_FeO_2_)(C_6_H_5_)_3_], the Sn^IV^ atom displays a distorted tetrahedral coordination geometry, provided by one O atom of the monodentate ferrocenecarboxylato ligand [Sn-O2 = 2.079 (2) Å] and by three C atoms of the three phenyl groups [average Sn-C = 2.130 (4) Å]. Bond lengths and angles involving the Sn metal centre are typical and comparable with those observed in related Sn^IV^ complexes (Zheng, Ma, Su *et al.*, 2004; Zheng, Ma, Yang *et al.*, 2004; Wang *et al.*, 2007).

No classic hydrogen bonds or intermolecular interactions are observed in the crystal.

### Experimental

To a stirred methanol (15 ml) solution of ferrocenecarboxyl sodium (1 mmol, 252 mg) was added triphenylchlorotin(1 mmol, 385 mg). The resulting red solution was stirred for 6 h and then the filtratation was allowed to stand at room temperature for about one week, whereupon red block crystals suitable for an X-ray diffraction analysis were obtained.

### Refinement

All H atoms were placed in geometrically idealized positions (C—H 0.93 Å) and treated as riding on their parent atoms, with *U*_iso_(H) = 1.2*U*_eq_(C).

### Crystal data

**Table:**

[FeSn(C_5_H_5_)(C_6_H_5_)_3_(C_6_H_4_O_2_)]	*Z* = 2
*M**_r_* = 579.02	*F*(000) = 580
Triclinic, *P*1	*D*_x_ = 1.593 Mg m^−^^3^
*a* = 10.1012 (15) Å	Mo *K*α radiation, λ = 0.71073 Å
*b* = 11.4066 (18) Å	Cell parameters from 3523 reflections
*c* = 11.6741 (19) Å	θ = 2.4–26.9°
α = 98.034 (1)°	µ = 1.66 mm^−^^1^
β = 106.107 (2)°	*T* = 298 K
γ = 106.082 (2)°	Block, red
*V* = 1207.1 (3) Å^3^	0.50 × 0.40 × 0.38 mm

### Data collection

**Table:**

Bruker SMART-1000 CCD diffractometer	4182 independent reflections
Radiation source: fine-focus sealed tube	3539 reflections with *I* > 2σ(*I*)
graphite	*R*_int_ = 0.021
phi and ω scans	θ_max_ = 25.0°, θ_min_ = 1.9°
Absorption correction: multi-scan (*SADABS*; Sheldrick, 1996)	*h* = −11→12
*T*_min_ = 0.491, *T*_max_ = 0.571	*k* = −13→13
6299 measured reflections	*l* = −13→10

### Refinement

**Table:**

Refinement on *F*^2^	Primary atom site location: structure-invariant direct methods
Least-squares matrix: full	Secondary atom site location: difference Fourier map
*R*[*F*^2^ > 2σ(*F*^2^)] = 0.030	Hydrogen site location: inferred from neighbouring sites
*wR*(*F*^2^) = 0.089	H-atom parameters constrained
*S* = 1.01	*w* = 1/[σ^2^(*F*_o_^2^) + (0.0548*P*)^2^ + 0.1113*P*] where *P* = (*F*_o_^2^ + 2*F*_c_^2^)/3
4182 reflections	(Δ/σ)_max_ < 0.001
298 parameters	Δρ_max_ = 0.46 e Å^−^^3^
0 restraints	Δρ_min_ = −0.70 e Å^−^^3^

### Fractional atomic coordinates and isotropic or equivalent isotropic displacement parameters (Å^2^)

**Table:**

	*x*	*y*	*z*	*U*_iso_*/*U*_eq_	
Sn	0.15862 (3)	0.36001 (2)	0.13590 (2)	0.03576 (11)	
Fe	−0.30164 (6)	0.22737 (5)	0.30190 (5)	0.04190 (15)	
O1	−0.0847 (3)	0.1793 (3)	0.0970 (3)	0.0491 (7)	
O2	−0.0337 (3)	0.3838 (2)	0.1496 (3)	0.0443 (6)	
C19	−0.1220 (4)	0.2694 (4)	0.1280 (3)	0.0375 (8)	
C1	0.1397 (4)	0.2675 (3)	−0.0417 (3)	0.0356 (8)	
C7	0.2778 (4)	0.5534 (3)	0.1604 (3)	0.0380 (8)	
C20	−0.2642 (4)	0.2547 (4)	0.1441 (3)	0.0413 (9)	
C13	0.2359 (4)	0.2953 (4)	0.2959 (3)	0.0396 (8)	
C2	0.1279 (4)	0.1420 (4)	−0.0740 (4)	0.0453 (9)	
H8	0.1325	0.0950	−0.0149	0.054*	
C6	0.1335 (5)	0.3349 (4)	−0.1312 (4)	0.0474 (10)	
H9	0.1440	0.4195	−0.1109	0.057*	
C21	−0.3748 (4)	0.1358 (4)	0.1220 (4)	0.0514 (10)	
H10	−0.3720	0.0531	0.0889	0.062*	
C27	−0.3368 (6)	0.1369 (5)	0.4353 (4)	0.0729 (15)	
H11	−0.4274	0.0715	0.4278	0.087*	
C4	0.0994 (5)	0.1542 (4)	−0.2809 (4)	0.0559 (11)	
H12	0.0840	0.1157	−0.3613	0.067*	
C17	0.2592 (5)	0.1324 (5)	0.4016 (5)	0.0677 (13)	
H13	0.2453	0.0479	0.3993	0.081*	
C23	−0.4528 (5)	0.2901 (5)	0.1981 (5)	0.0666 (14)	
H14	−0.5132	0.3318	0.2287	0.080*	
C3	0.1096 (5)	0.0867 (4)	−0.1922 (4)	0.0528 (11)	
H15	0.1041	0.0033	−0.2122	0.063*	
C22	−0.4898 (5)	0.1617 (5)	0.1555 (4)	0.0655 (13)	
H16	−0.5803	0.0985	0.1515	0.079*	
C8	0.4041 (4)	0.5854 (4)	0.1286 (4)	0.0533 (11)	
H17	0.4362	0.5219	0.0999	0.064*	
C5	0.1118 (5)	0.2781 (4)	−0.2511 (4)	0.0589 (11)	
H18	0.1057	0.3241	−0.3110	0.071*	
C18	0.2145 (4)	0.1700 (4)	0.2944 (4)	0.0497 (10)	
H19	0.1694	0.1104	0.2201	0.060*	
C24	−0.3112 (5)	0.3517 (4)	0.1938 (5)	0.0584 (12)	
H20	−0.2580	0.4421	0.2178	0.070*	
C12	0.2319 (5)	0.6496 (4)	0.2018 (4)	0.0571 (11)	
H21	0.1477	0.6314	0.2234	0.069*	
C10	0.4356 (6)	0.8009 (5)	0.1798 (4)	0.0708 (15)	
H22	0.4883	0.8841	0.1869	0.085*	
C14	0.3036 (5)	0.3814 (4)	0.4074 (4)	0.0586 (11)	
H23	0.3204	0.4664	0.4106	0.070*	
C16	0.3243 (6)	0.2196 (6)	0.5123 (5)	0.0786 (16)	
H24	0.3525	0.1939	0.5848	0.094*	
C28	−0.3050 (7)	0.2640 (5)	0.4773 (5)	0.0752 (15)	
H25	−0.3685	0.3039	0.5051	0.090*	
C25	−0.1146 (6)	0.2310 (8)	0.4244 (5)	0.093 (2)	
H26	−0.0200	0.2445	0.4119	0.111*	
C26	−0.2222 (8)	0.1166 (7)	0.4036 (5)	0.0824 (17)	
H27	−0.2176	0.0344	0.3709	0.099*	
C29	−0.1673 (7)	0.3262 (6)	0.4719 (5)	0.0877 (18)	
H28	−0.1155	0.4169	0.4972	0.105*	
C9	0.4823 (5)	0.7075 (5)	0.1385 (4)	0.0656 (13)	
H29	0.5666	0.7265	0.1171	0.079*	
C15	0.3470 (6)	0.3421 (6)	0.5152 (4)	0.0793 (16)	
H30	0.3921	0.4010	0.5899	0.095*	
C11	0.3109 (7)	0.7730 (4)	0.2113 (5)	0.0798 (16)	
H32	0.2795	0.8374	0.2391	0.096*	

### Atomic displacement parameters (Å^2^)

**Table:**

	*U*^11^	*U*^22^	*U*^33^	*U*^12^	*U*^13^	*U*^23^
Sn	0.03738 (16)	0.03568 (16)	0.03835 (16)	0.01117 (11)	0.01736 (11)	0.01373 (11)
Fe	0.0444 (3)	0.0463 (3)	0.0446 (3)	0.0188 (3)	0.0219 (3)	0.0183 (3)
O1	0.0521 (16)	0.0472 (16)	0.0579 (18)	0.0190 (13)	0.0270 (14)	0.0197 (13)
O2	0.0395 (14)	0.0443 (16)	0.0595 (17)	0.0157 (12)	0.0253 (13)	0.0227 (13)
C19	0.0342 (19)	0.048 (2)	0.035 (2)	0.0132 (17)	0.0141 (16)	0.0203 (17)
C1	0.0385 (19)	0.039 (2)	0.0353 (19)	0.0154 (16)	0.0166 (16)	0.0121 (16)
C7	0.0384 (19)	0.036 (2)	0.036 (2)	0.0071 (16)	0.0099 (16)	0.0124 (16)
C20	0.0360 (19)	0.050 (2)	0.040 (2)	0.0125 (17)	0.0123 (16)	0.0239 (18)
C13	0.0349 (19)	0.051 (2)	0.039 (2)	0.0159 (17)	0.0163 (16)	0.0152 (17)
C2	0.053 (2)	0.040 (2)	0.051 (2)	0.0182 (18)	0.0224 (19)	0.0200 (18)
C6	0.064 (3)	0.038 (2)	0.044 (2)	0.0186 (19)	0.021 (2)	0.0153 (18)
C21	0.044 (2)	0.059 (3)	0.043 (2)	0.003 (2)	0.0133 (19)	0.017 (2)
C27	0.107 (4)	0.079 (4)	0.056 (3)	0.037 (3)	0.047 (3)	0.034 (3)
C4	0.066 (3)	0.054 (3)	0.044 (2)	0.018 (2)	0.020 (2)	0.003 (2)
C17	0.068 (3)	0.073 (3)	0.069 (3)	0.024 (3)	0.022 (3)	0.041 (3)
C23	0.047 (2)	0.092 (4)	0.093 (4)	0.040 (3)	0.040 (3)	0.055 (3)
C3	0.064 (3)	0.038 (2)	0.056 (3)	0.013 (2)	0.026 (2)	0.0052 (19)
C22	0.038 (2)	0.090 (4)	0.065 (3)	0.012 (2)	0.017 (2)	0.029 (3)
C8	0.046 (2)	0.053 (3)	0.057 (3)	0.008 (2)	0.018 (2)	0.017 (2)
C5	0.081 (3)	0.061 (3)	0.043 (2)	0.029 (2)	0.021 (2)	0.023 (2)
C18	0.052 (2)	0.054 (3)	0.047 (2)	0.018 (2)	0.018 (2)	0.022 (2)
C24	0.058 (3)	0.062 (3)	0.078 (3)	0.030 (2)	0.034 (2)	0.045 (2)
C12	0.064 (3)	0.046 (3)	0.061 (3)	0.016 (2)	0.024 (2)	0.009 (2)
C10	0.076 (3)	0.048 (3)	0.055 (3)	−0.013 (3)	0.002 (3)	0.018 (2)
C14	0.061 (3)	0.061 (3)	0.048 (3)	0.015 (2)	0.016 (2)	0.008 (2)
C16	0.064 (3)	0.112 (5)	0.059 (3)	0.022 (3)	0.013 (3)	0.051 (3)
C28	0.095 (4)	0.086 (4)	0.056 (3)	0.036 (3)	0.040 (3)	0.010 (3)
C25	0.063 (3)	0.189 (7)	0.043 (3)	0.057 (4)	0.018 (3)	0.046 (4)
C26	0.120 (5)	0.117 (5)	0.058 (3)	0.081 (4)	0.046 (3)	0.049 (3)
C29	0.083 (4)	0.093 (4)	0.055 (3)	0.005 (3)	0.009 (3)	−0.001 (3)
C9	0.054 (3)	0.068 (3)	0.062 (3)	−0.004 (2)	0.017 (2)	0.027 (3)
C15	0.074 (3)	0.105 (5)	0.037 (3)	0.013 (3)	0.006 (2)	0.010 (3)
C11	0.110 (4)	0.041 (3)	0.079 (4)	0.022 (3)	0.024 (3)	0.007 (2)

### Geometric parameters (Å, °)

**Table:**

Sn—O2	2.079 (2)	C4—C3	1.372 (6)
Sn—C1	2.123 (3)	C4—H12	0.9300
Sn—C13	2.131 (4)	C17—C16	1.378 (7)
Sn—C7	2.136 (4)	C17—C18	1.378 (6)
Fe—C25	2.014 (5)	C17—H13	0.9300
Fe—C29	2.021 (5)	C23—C22	1.386 (7)
Fe—C26	2.026 (5)	C23—C24	1.427 (6)
Fe—C20	2.029 (4)	C23—H14	0.9800
Fe—C24	2.030 (4)	C3—H15	0.9300
Fe—C23	2.033 (4)	C22—H16	0.9800
Fe—C21	2.036 (4)	C8—C9	1.368 (6)
Fe—C22	2.036 (5)	C8—H17	0.9300
Fe—C27	2.042 (5)	C5—H18	0.9300
Fe—C28	2.043 (5)	C18—H19	0.9300
O1—C19	1.231 (4)	C24—H20	0.9800
O2—C19	1.304 (4)	C12—C11	1.384 (7)
C19—C20	1.467 (5)	C12—H21	0.9300
C1—C6	1.378 (5)	C10—C9	1.362 (7)
C1—C2	1.392 (5)	C10—C11	1.374 (7)
C7—C12	1.382 (6)	C10—H22	0.9300
C7—C8	1.393 (5)	C14—C15	1.392 (7)
C20—C24	1.431 (6)	C14—H23	0.9300
C20—C21	1.435 (5)	C16—C15	1.345 (8)
C13—C14	1.380 (5)	C16—H24	0.9300
C13—C18	1.382 (6)	C28—C29	1.399 (8)
C2—C3	1.373 (6)	C28—H25	0.9800
C2—H8	0.9300	C25—C26	1.387 (9)
C6—C5	1.386 (6)	C25—C29	1.444 (9)
C6—H9	0.9300	C25—H26	0.9800
C21—C22	1.414 (6)	C26—H27	0.9800
C21—H10	0.9800	C29—H28	0.9800
C27—C26	1.376 (7)	C9—H29	0.9300
C27—C28	1.379 (7)	C15—H30	0.9300
C27—H11	0.9800	C11—H32	0.9300
C4—C5	1.370 (6)		
			
O2—Sn—C1	114.16 (12)	C26—C27—H11	125.4
O2—Sn—C13	102.45 (12)	C28—C27—H11	125.4
C1—Sn—C13	122.73 (14)	Fe—C27—H11	125.4
O2—Sn—C7	97.22 (12)	C5—C4—C3	120.0 (4)
C1—Sn—C7	106.57 (14)	C5—C4—H12	120.0
C13—Sn—C7	110.83 (14)	C3—C4—H12	120.0
C25—Fe—C29	41.9 (3)	C16—C17—C18	120.2 (5)
C25—Fe—C26	40.2 (3)	C16—C17—H13	119.9
C29—Fe—C26	68.2 (3)	C18—C17—H13	119.9
C25—Fe—C20	108.67 (19)	C22—C23—C24	109.4 (4)
C29—Fe—C20	124.7 (2)	C22—C23—Fe	70.2 (3)
C26—Fe—C20	124.14 (19)	C24—C23—Fe	69.3 (2)
C25—Fe—C24	123.3 (3)	C22—C23—H14	125.3
C29—Fe—C24	107.1 (2)	C24—C23—H14	125.3
C26—Fe—C24	159.9 (2)	Fe—C23—H14	125.3
C20—Fe—C24	41.31 (17)	C4—C3—C2	120.3 (4)
C25—Fe—C23	159.7 (3)	C4—C3—H15	119.9
C29—Fe—C23	122.0 (3)	C2—C3—H15	119.9
C26—Fe—C23	158.1 (3)	C23—C22—C21	109.4 (4)
C20—Fe—C23	68.39 (17)	C23—C22—Fe	70.0 (3)
C24—Fe—C23	41.12 (17)	C21—C22—Fe	69.7 (2)
C25—Fe—C21	123.7 (2)	C23—C22—H16	125.3
C29—Fe—C21	161.7 (2)	C21—C22—H16	125.3
C26—Fe—C21	108.1 (2)	Fe—C22—H16	125.3
C20—Fe—C21	41.33 (15)	C9—C8—C7	121.9 (4)
C24—Fe—C21	69.90 (19)	C9—C8—H17	119.0
C23—Fe—C21	68.3 (2)	C7—C8—H17	119.0
C25—Fe—C22	159.5 (3)	C4—C5—C6	119.8 (4)
C29—Fe—C22	156.5 (3)	C4—C5—H18	120.1
C26—Fe—C22	123.5 (3)	C6—C5—H18	120.1
C20—Fe—C22	68.30 (16)	C17—C18—C13	120.7 (4)
C24—Fe—C22	68.7 (2)	C17—C18—H19	119.7
C23—Fe—C22	39.8 (2)	C13—C18—H19	119.7
C21—Fe—C22	40.62 (18)	C23—C24—C20	106.0 (4)
C25—Fe—C27	67.4 (2)	C23—C24—Fe	69.6 (2)
C29—Fe—C27	67.3 (2)	C20—C24—Fe	69.3 (2)
C26—Fe—C27	39.5 (2)	C23—C24—H20	127.0
C20—Fe—C27	158.90 (19)	C20—C24—H20	127.0
C24—Fe—C27	158.5 (2)	Fe—C24—H20	127.0
C23—Fe—C27	122.7 (2)	C7—C12—C11	120.1 (5)
C21—Fe—C27	122.1 (2)	C7—C12—H21	119.9
C22—Fe—C27	107.7 (2)	C11—C12—H21	119.9
C25—Fe—C28	68.5 (2)	C9—C10—C11	120.4 (4)
C29—Fe—C28	40.3 (2)	C9—C10—H22	119.8
C26—Fe—C28	67.0 (2)	C11—C10—H22	119.8
C20—Fe—C28	160.6 (2)	C13—C14—C15	120.5 (5)
C24—Fe—C28	122.9 (2)	C13—C14—H23	119.8
C23—Fe—C28	107.2 (2)	C15—C14—H23	119.8
C21—Fe—C28	156.5 (2)	C15—C16—C17	119.8 (5)
C22—Fe—C28	121.2 (2)	C15—C16—H24	120.1
C27—Fe—C28	39.5 (2)	C17—C16—H24	120.1
C19—O2—Sn	103.8 (2)	C27—C28—C29	108.2 (5)
O1—C19—O2	120.4 (3)	C27—C28—Fe	70.2 (3)
O1—C19—C20	122.7 (3)	C29—C28—Fe	69.0 (3)
O2—C19—C20	116.9 (3)	C27—C28—H25	125.9
C6—C1—C2	118.2 (3)	C29—C28—H25	125.9
C6—C1—Sn	117.9 (3)	Fe—C28—H25	125.9
C2—C1—Sn	123.8 (3)	C26—C25—C29	106.5 (5)
C12—C7—C8	117.8 (4)	C26—C25—Fe	70.4 (3)
C12—C7—Sn	122.8 (3)	C29—C25—Fe	69.3 (3)
C8—C7—Sn	119.3 (3)	C26—C25—H26	126.7
C24—C20—C21	108.7 (3)	C29—C25—H26	126.7
C24—C20—C19	127.0 (4)	Fe—C25—H26	126.7
C21—C20—C19	124.0 (4)	C27—C26—C25	109.2 (5)
C24—C20—Fe	69.4 (2)	C27—C26—Fe	70.9 (3)
C21—C20—Fe	69.6 (2)	C25—C26—Fe	69.5 (3)
C19—C20—Fe	122.6 (2)	C27—C26—H27	125.4
C14—C13—C18	118.2 (4)	C25—C26—H27	125.4
C14—C13—Sn	118.6 (3)	Fe—C26—H27	125.4
C18—C13—Sn	123.0 (3)	C28—C29—C25	106.9 (6)
C3—C2—C1	120.7 (4)	C28—C29—Fe	70.7 (3)
C3—C2—H8	119.6	C25—C29—Fe	68.8 (3)
C1—C2—H8	119.6	C28—C29—H28	126.5
C1—C6—C5	120.9 (4)	C25—C29—H28	126.5
C1—C6—H9	119.5	Fe—C29—H28	126.5
C5—C6—H9	119.5	C10—C9—C8	119.4 (5)
C22—C21—C20	106.5 (4)	C10—C9—H29	120.3
C22—C21—Fe	69.7 (3)	C8—C9—H29	120.3
C20—C21—Fe	69.0 (2)	C16—C15—C14	120.6 (5)
C22—C21—H10	126.8	C16—C15—H30	119.7
C20—C21—H10	126.8	C14—C15—H30	119.7
Fe—C21—H10	126.8	C10—C11—C12	120.3 (5)
C26—C27—C28	109.2 (6)	C10—C11—H32	119.8
C26—C27—Fe	69.6 (3)	C12—C11—H32	119.8
C28—C27—Fe	70.3 (3)		
			
C1—Sn—O2—C19	64.2 (2)	C29—Fe—C22—C21	167.5 (5)
C13—Sn—O2—C19	−70.7 (2)	C26—Fe—C22—C21	−78.5 (3)
C7—Sn—O2—C19	176.0 (2)	C20—Fe—C22—C21	39.0 (3)
Sn—O2—C19—O1	−5.6 (4)	C24—Fe—C22—C21	83.5 (3)
Sn—O2—C19—C20	173.6 (3)	C23—Fe—C22—C21	120.8 (4)
O2—Sn—C1—C6	75.2 (3)	C27—Fe—C22—C21	−119.0 (3)
C13—Sn—C1—C6	−160.1 (3)	C28—Fe—C22—C21	−160.0 (3)
C7—Sn—C1—C6	−30.9 (3)	C12—C7—C8—C9	0.6 (6)
O2—Sn—C1—C2	−102.4 (3)	Sn—C7—C8—C9	177.8 (3)
C13—Sn—C1—C2	22.3 (4)	C3—C4—C5—C6	−0.5 (7)
C7—Sn—C1—C2	151.5 (3)	C1—C6—C5—C4	−1.4 (7)
O2—Sn—C7—C12	13.0 (4)	C16—C17—C18—C13	0.9 (7)
C1—Sn—C7—C12	130.9 (3)	C14—C13—C18—C17	0.3 (6)
C13—Sn—C7—C12	−93.3 (4)	Sn—C13—C18—C17	−176.2 (3)
O2—Sn—C7—C8	−164.1 (3)	C22—C23—C24—C20	1.1 (5)
C1—Sn—C7—C8	−46.2 (3)	Fe—C23—C24—C20	60.1 (3)
C13—Sn—C7—C8	89.6 (3)	C22—C23—C24—Fe	−58.9 (3)
O1—C19—C20—C24	171.8 (4)	C21—C20—C24—C23	−1.6 (5)
O2—C19—C20—C24	−7.4 (6)	C19—C20—C24—C23	−176.2 (4)
O1—C19—C20—C21	−2.1 (6)	Fe—C20—C24—C23	−60.2 (3)
O2—C19—C20—C21	178.7 (3)	C21—C20—C24—Fe	58.7 (3)
O1—C19—C20—Fe	84.1 (4)	C19—C20—C24—Fe	−116.0 (4)
O2—C19—C20—Fe	−95.1 (4)	C25—Fe—C24—C23	−162.5 (4)
C25—Fe—C20—C24	−119.5 (3)	C29—Fe—C24—C23	−119.3 (4)
C29—Fe—C20—C24	−75.7 (4)	C26—Fe—C24—C23	167.6 (6)
C26—Fe—C20—C24	−161.3 (3)	C20—Fe—C24—C23	117.1 (4)
C23—Fe—C20—C24	39.0 (3)	C21—Fe—C24—C23	79.7 (3)
C21—Fe—C20—C24	120.4 (3)	C22—Fe—C24—C23	36.2 (3)
C22—Fe—C20—C24	82.0 (3)	C27—Fe—C24—C23	−48.1 (7)
C27—Fe—C20—C24	164.9 (5)	C28—Fe—C24—C23	−78.1 (4)
C28—Fe—C20—C24	−41.3 (7)	C25—Fe—C24—C20	80.4 (3)
C25—Fe—C20—C21	120.1 (3)	C29—Fe—C24—C20	123.6 (3)
C29—Fe—C20—C21	164.0 (3)	C26—Fe—C24—C20	50.5 (8)
C26—Fe—C20—C21	78.3 (4)	C23—Fe—C24—C20	−117.1 (4)
C24—Fe—C20—C21	−120.4 (3)	C21—Fe—C24—C20	−37.4 (2)
C23—Fe—C20—C21	−81.3 (3)	C22—Fe—C24—C20	−80.9 (3)
C22—Fe—C20—C21	−38.3 (3)	C27—Fe—C24—C20	−165.2 (5)
C27—Fe—C20—C21	44.5 (6)	C28—Fe—C24—C20	164.8 (3)
C28—Fe—C20—C21	−161.6 (6)	C8—C7—C12—C11	−0.3 (6)
C25—Fe—C20—C19	2.0 (4)	Sn—C7—C12—C11	−177.5 (4)
C29—Fe—C20—C19	45.9 (4)	C18—C13—C14—C15	−0.9 (6)
C26—Fe—C20—C19	−39.8 (4)	Sn—C13—C14—C15	175.7 (4)
C24—Fe—C20—C19	121.6 (4)	C18—C17—C16—C15	−1.4 (8)
C23—Fe—C20—C19	160.6 (4)	C26—C27—C28—C29	0.2 (6)
C21—Fe—C20—C19	−118.1 (4)	Fe—C27—C28—C29	−58.7 (4)
C22—Fe—C20—C19	−156.4 (4)	C26—C27—C28—Fe	58.9 (4)
C27—Fe—C20—C19	−73.5 (7)	C25—Fe—C28—C27	−80.1 (4)
C28—Fe—C20—C19	80.3 (7)	C29—Fe—C28—C27	−119.6 (5)
O2—Sn—C13—C14	−77.8 (3)	C26—Fe—C28—C27	−36.6 (4)
C1—Sn—C13—C14	152.4 (3)	C20—Fe—C28—C27	−165.5 (5)
C7—Sn—C13—C14	25.0 (3)	C24—Fe—C28—C27	163.2 (3)
O2—Sn—C13—C18	98.6 (3)	C23—Fe—C28—C27	120.9 (4)
C1—Sn—C13—C18	−31.2 (4)	C21—Fe—C28—C27	45.9 (7)
C7—Sn—C13—C18	−158.6 (3)	C22—Fe—C28—C27	79.7 (4)
C6—C1—C2—C3	−0.4 (6)	C25—Fe—C28—C29	39.4 (4)
Sn—C1—C2—C3	177.1 (3)	C26—Fe—C28—C29	82.9 (4)
C2—C1—C6—C5	1.9 (6)	C20—Fe—C28—C29	−46.0 (8)
Sn—C1—C6—C5	−175.9 (3)	C24—Fe—C28—C29	−77.2 (4)
C24—C20—C21—C22	1.4 (5)	C23—Fe—C28—C29	−119.5 (4)
C19—C20—C21—C22	176.3 (4)	C21—Fe—C28—C29	165.5 (5)
Fe—C20—C21—C22	59.9 (3)	C22—Fe—C28—C29	−160.7 (4)
C24—C20—C21—Fe	−58.5 (3)	C27—Fe—C28—C29	119.6 (5)
C19—C20—C21—Fe	116.3 (3)	C29—Fe—C25—C26	117.2 (5)
C25—Fe—C21—C22	162.3 (4)	C20—Fe—C25—C26	−121.2 (3)
C29—Fe—C21—C22	−164.1 (7)	C24—Fe—C25—C26	−164.6 (3)
C26—Fe—C21—C22	120.8 (3)	C23—Fe—C25—C26	160.8 (5)
C20—Fe—C21—C22	−117.7 (4)	C21—Fe—C25—C26	−77.9 (3)
C24—Fe—C21—C22	−80.4 (3)	C22—Fe—C25—C26	−43.4 (7)
C23—Fe—C21—C22	−36.3 (3)	C27—Fe—C25—C26	36.6 (3)
C27—Fe—C21—C22	79.6 (4)	C28—Fe—C25—C26	79.3 (3)
C28—Fe—C21—C22	47.0 (6)	C26—Fe—C25—C29	−117.2 (5)
C25—Fe—C21—C20	−79.9 (4)	C20—Fe—C25—C29	121.6 (3)
C29—Fe—C21—C20	−46.3 (8)	C24—Fe—C25—C29	78.2 (4)
C26—Fe—C21—C20	−121.5 (3)	C23—Fe—C25—C29	43.5 (7)
C24—Fe—C21—C20	37.3 (2)	C21—Fe—C25—C29	164.9 (3)
C23—Fe—C21—C20	81.5 (3)	C22—Fe—C25—C29	−160.6 (5)
C22—Fe—C21—C20	117.7 (4)	C27—Fe—C25—C29	−80.6 (4)
C27—Fe—C21—C20	−162.7 (3)	C28—Fe—C25—C29	−37.9 (4)
C28—Fe—C21—C20	164.8 (5)	C28—C27—C26—C25	−0.3 (6)
C25—Fe—C27—C26	−37.2 (4)	Fe—C27—C26—C25	59.1 (4)
C29—Fe—C27—C26	−82.8 (4)	C28—C27—C26—Fe	−59.3 (4)
C20—Fe—C27—C26	46.3 (7)	C29—C25—C26—C27	0.2 (6)
C24—Fe—C27—C26	−161.7 (6)	Fe—C25—C26—C27	−59.9 (4)
C23—Fe—C27—C26	162.8 (4)	C29—C25—C26—Fe	60.2 (4)
C21—Fe—C27—C26	79.4 (4)	C25—Fe—C26—C27	120.1 (5)
C22—Fe—C27—C26	121.6 (4)	C29—Fe—C26—C27	80.3 (4)
C28—Fe—C27—C26	−120.4 (6)	C20—Fe—C26—C27	−161.7 (3)
C25—Fe—C27—C28	83.2 (4)	C24—Fe—C26—C27	160.4 (6)
C29—Fe—C27—C28	37.6 (4)	C23—Fe—C26—C27	−42.0 (8)
C26—Fe—C27—C28	120.4 (6)	C21—Fe—C26—C27	−118.8 (4)
C20—Fe—C27—C28	166.6 (5)	C22—Fe—C26—C27	−76.7 (4)
C24—Fe—C27—C28	−41.3 (8)	C28—Fe—C26—C27	36.6 (3)
C23—Fe—C27—C28	−76.8 (4)	C29—Fe—C26—C25	−39.8 (3)
C21—Fe—C27—C28	−160.2 (3)	C20—Fe—C26—C25	78.2 (4)
C22—Fe—C27—C28	−118.0 (4)	C24—Fe—C26—C25	40.3 (8)
C25—Fe—C23—C22	167.1 (5)	C23—Fe—C26—C25	−162.1 (6)
C29—Fe—C23—C22	−160.0 (3)	C21—Fe—C26—C25	121.1 (3)
C26—Fe—C23—C22	−47.7 (7)	C22—Fe—C26—C25	163.3 (3)
C20—Fe—C23—C22	81.6 (3)	C27—Fe—C26—C25	−120.1 (5)
C24—Fe—C23—C22	120.8 (4)	C28—Fe—C26—C25	−83.5 (4)
C21—Fe—C23—C22	37.0 (3)	C27—C28—C29—C25	−0.1 (6)
C27—Fe—C23—C22	−78.1 (4)	Fe—C28—C29—C25	−59.5 (4)
C28—Fe—C23—C22	−118.5 (3)	C27—C28—C29—Fe	59.5 (4)
C25—Fe—C23—C24	46.3 (7)	C26—C25—C29—C28	−0.1 (6)
C29—Fe—C23—C24	79.2 (4)	Fe—C25—C29—C28	60.8 (4)
C26—Fe—C23—C24	−168.5 (5)	C26—C25—C29—Fe	−60.9 (3)
C20—Fe—C23—C24	−39.2 (3)	C25—Fe—C29—C28	−117.8 (6)
C21—Fe—C23—C24	−83.8 (3)	C26—Fe—C29—C28	−79.7 (4)
C22—Fe—C23—C24	−120.8 (4)	C20—Fe—C29—C28	163.1 (3)
C27—Fe—C23—C24	161.1 (3)	C24—Fe—C29—C28	121.1 (4)
C28—Fe—C23—C24	120.7 (3)	C23—Fe—C29—C28	78.6 (4)
C5—C4—C3—C2	2.0 (7)	C21—Fe—C29—C28	−161.4 (6)
C1—C2—C3—C4	−1.5 (6)	C22—Fe—C29—C28	45.3 (8)
C24—C23—C22—C21	−0.3 (6)	C27—Fe—C29—C28	−36.8 (4)
Fe—C23—C22—C21	−58.6 (3)	C26—Fe—C29—C25	38.1 (3)
C24—C23—C22—Fe	58.4 (3)	C20—Fe—C29—C25	−79.1 (4)
C20—C21—C22—C23	−0.7 (5)	C24—Fe—C29—C25	−121.1 (4)
Fe—C21—C22—C23	58.8 (3)	C23—Fe—C29—C25	−163.6 (4)
C20—C21—C22—Fe	−59.5 (3)	C21—Fe—C29—C25	−43.6 (9)
C25—Fe—C22—C23	−167.2 (6)	C22—Fe—C29—C25	163.1 (5)
C29—Fe—C22—C23	46.7 (7)	C27—Fe—C29—C25	81.0 (4)
C26—Fe—C22—C23	160.7 (3)	C28—Fe—C29—C25	117.8 (6)
C20—Fe—C22—C23	−81.9 (3)	C11—C10—C9—C8	−0.1 (7)
C24—Fe—C22—C23	−37.3 (3)	C7—C8—C9—C10	−0.4 (7)
C21—Fe—C22—C23	−120.8 (4)	C17—C16—C15—C14	0.8 (9)
C27—Fe—C22—C23	120.2 (3)	C13—C14—C15—C16	0.3 (8)
C28—Fe—C22—C23	79.2 (3)	C9—C10—C11—C12	0.3 (8)
C25—Fe—C22—C21	−46.4 (7)	C7—C12—C11—C10	−0.1 (7)
